# Higher remnant cholesterol to lymphocyte ratio is associated with vulnerable plaque features in acute coronary syndrome

**DOI:** 10.3389/fcvm.2026.1785669

**Published:** 2026-07-23

**Authors:** Yingzheng Liu, Xuesong Liu, Weiren Yan, Ronglin Sun, Yao Yu, Aiming Chen, Zhenyu Dong, Xinsheng Li, Haichen Lv

**Affiliations:** 1 The First People's Hospital of Jinzhou District, Affiliated to Dalian University, Dalian, Liaoning Province, China; 2 Department of Cardiology, the First Affiliated Hospital of Dalian Medical University, Dalian, Liaoning Province, China

**Keywords:** acute coronary syndrome, lymphocytes, optical coherence tomography, remnant cholesterol, vulnerable plaque

## Abstract

**Background:**

Remnant cholesterol to lymphocyte ratio (RCLR) integrates atherogenic lipid metabolism and systemic inflammation, potentially reflecting plaque vulnerability in acute coronary syndrome (ACS). However, its association with intracoronary imaging features remains unclear. This study aimed to investigate the relationship between RCLR and culprit plaque characteristics using optical coherence tomography (OCT).

**Methods:**

This retrospective study analyzed 450 ACS inpatients (2021–2024), with 201 included after rigorous selection. The patients were classified into three groups based on the remnant cholesterol to lymphocyte ratio [RCLR; remnant cholesterol (RC = TC−HDL-C−LDL-C) divided by lymphocyte percentage], using tertiles: Group T1 (RCLR ≤ 3.03, *n* = 66), Group T2 (3.03 < RCLR ≤ 4.66, *n* = 68), and Group T3 (RCLR > 4.66, *n* = 67). The characteristics of the culprit lesion plaques were assessed using OCT, and differences among the groups, the correlation between RCLR and plaque characteristics, and multivariable logistic regression were analyzed using SPSS version 27.0 and Python, with two-sided statistical significance set at *p* < 0.05 (with Bonferroni correction for *post-hoc* pairwise comparisons).

**Results:**

OCT imaging analysis revealed significant differences in qualitative features among various RCLR groups, including plaque rupture, lipid plaque, thin-cap fibroatheroma (TCFA), and superficial calcification (all *p* values < 0.05). In an exploratory analysis restricted to lesions containing lipid plaque, RCLR showed weak positive correlations with the maximum lipid arc (*r* = 0.252, 95% CI: 0.026–0.454, *p* = 0.022) and the lipid index (*r* = 0.279, 95% CI: 0.062–0.481, *p* = 0.011). Receiver operating characteristic (ROC) curve analysis further confirmed that RCLR had modest discriminatory value for lipid plaques (AUC = 0.635, 95% CI: 0.559–0.712, *p* = 0.001), TCFA (AUC = 0.646, 95% CI: 0.559–0.733, *p* = 0.003), and plaque rupture (AUC = 0.618, 95% CI: 0.540–0.696, *p* = 0.004). In multivariable analysis, the highest RCLR tertile remained associated with lipid-rich plaque, TCFA, and plaque rupture after adjustment for major clinical risk factors (all P for trend ≤ 0.011).

**Conclusion:**

In ACS patients, a higher RCLR was associated with high-risk plaque characteristics detected by OCT, such as lipid-rich plaques, TCFA, and plaque rupture, and the highest RCLR tertile remained associated with these features after multivariable adjustment. RCLR exhibits a certain predictive ability for these vulnerable plaque features. Higher RCLR tertiles were associated with a greater prevalence of vulnerable plaque features, suggesting that RCLR may serve as a composite lipid–inflammatory marker associated with coronary plaque vulnerability, although its signal partly overlaps with systemic inflammatory-cell parameters.

## Introduction

Coronary artery disease (CAD) has emerged as the leading cause of death among the Chinese population (1). Dyslipidemia is a well-established risk factor for CAD, and over the past few decades, blood lipids have been recognized as a crucial target in both the prevention and treatment of this condition (2–5). Various types of studies, including observational and genetic research, as well as randomized controlled trials, have demonstrated that despite achieving target levels of low-density lipoprotein cholesterol (LDL-c), some patients continue to experience recurrent major adverse cardiovascular events (MACE) (6). Recent studies have indicated that high levels of remnant cholesterol (RC) may significantly contribute to the risk of remnant disease (7, 8). Furthermore, inflammation plays a pivotal role in the development of CAD (9–11), and the peripheral blood lymphocyte count serves as a reliable indicator of the body's inflammatory status. Previous studies have shown that a reduced lymphocyte count is associated with adverse outcomes in cardiovascular diseases (12–15).

Recent research has suggested that the Remnant Cholesterol to Lymphocyte Ratio (RCLR) is a strong predictor of MACE (16), as this metric accounts for both atherosclerosis and inflammatory factors. Currently, there is no research investigating the correlation between RCLR and the characteristics of intracoronary plaques in patients with acute coronary syndrome (ACS). Optical Coherence Tomography (OCT) provides extremely high resolution, allowing for clear visualization of the coronary intima and plaque components, thereby revealing the morphological characteristics of atherosclerotic plaques.

Consequently, this study aims to utilize OCT to assess the relationship between RCLR and the characteristics of culprit coronary plaques in patients with ACS.

## Methods

### Study design and population

**Figure 1:**
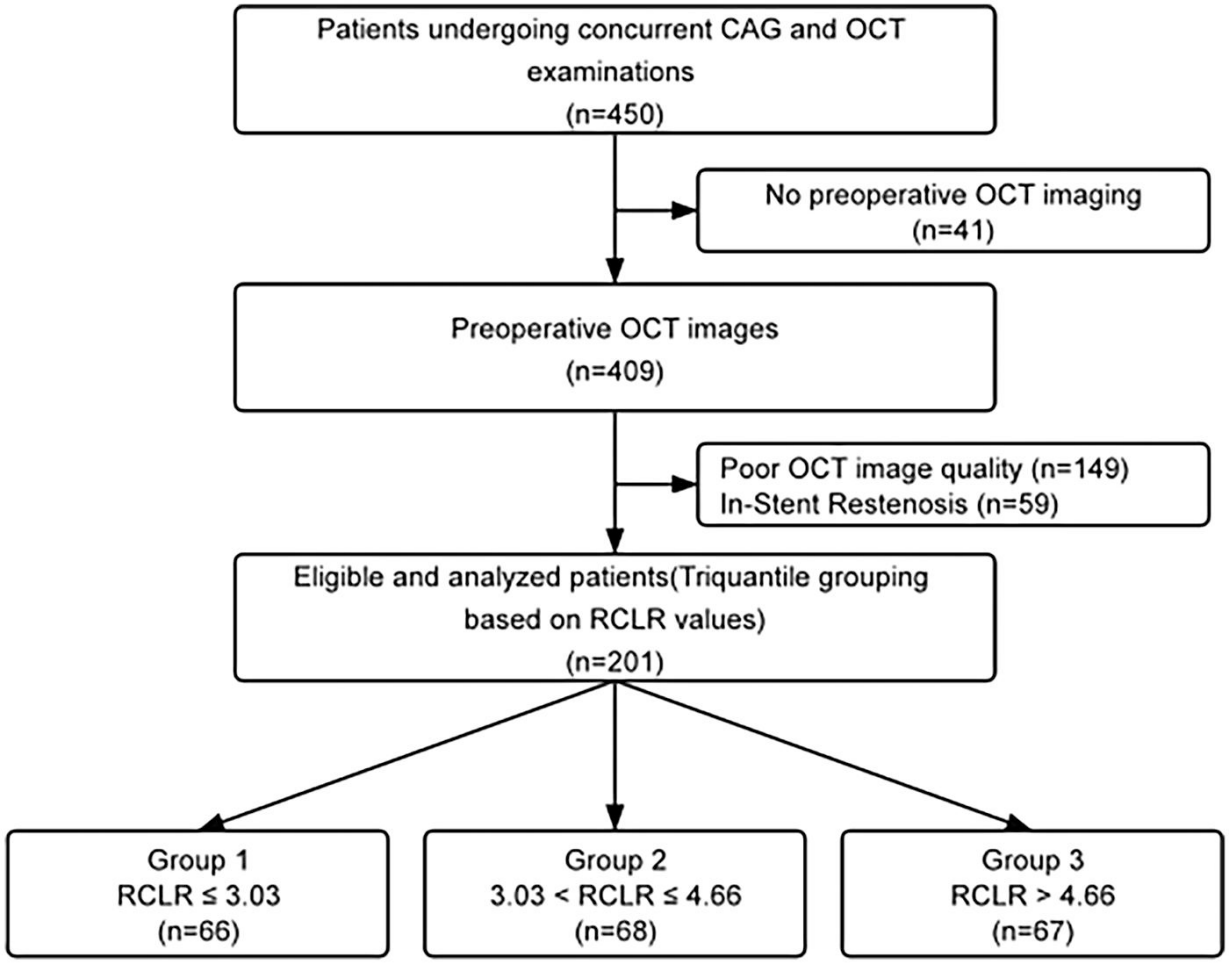
Study flow chart. CAG, coronary angiography; RCLR, remnant cholesterol to lymphocyte ratio.

This study employed a retrospective search strategy to enroll 450 patients diagnosed with ACS who were hospitalized in the Department of Cardiology at the First Affiliated Hospital of Dalian Medical University between January 2021 and June 2024. All participants underwent both coronary angiography and OCT. The diagnosis of ACS adhered strictly to the latest ACS guidelines (17). Demographic data, past medical history, and relevant laboratory results were meticulously recorded. Inclusion criteria for the study comprised an ACS diagnosis based on chest pain symptoms, elevated high-sensitivity troponin I levels, and electrocardiogram findings. Informed consent for surgical procedures was obtained upon admission, and OCT was performed concurrently with coronary angiography and interventional therapy. A total of 149 patients with poor OCT image quality were excluded, along with 59 patients who experienced restenosis after coronary stenting and 41 patients who did not undergo OCT examination prior to the procedure, resulting in a final inclusion of 201 patients (corresponding to 201 lesions),as shown in Figure 1. The baseline characteristics of the included and the excluded patients are compared in Supplementary Table S1. This study received approval from the Ethics Committee of Dalian Medical University, and all patients' personal information was kept strictly confidential.

### Data collection and OCT image measurement

**Figure 2:**
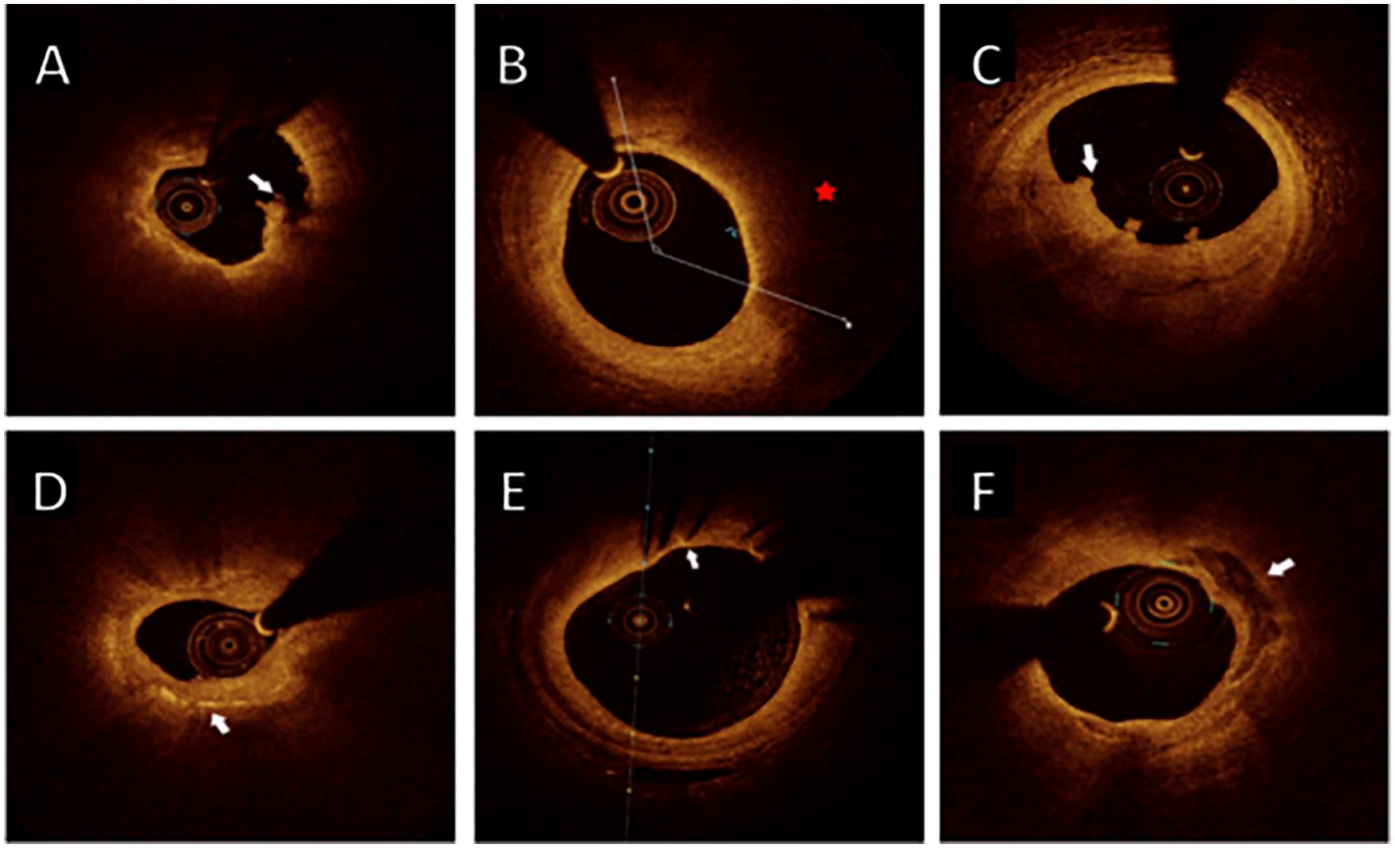
The typical OCT images. **(A)** Rupture of plaque with cavity formation. **(B)** TCFA,the red five-pointed star represents a massive lipid pool. **(C)** Plaque erosion, no fracture of the fibrous cap observed, with white thrombus present. **(D)** Cholesterol crystal **(E)** Macrophage **(F)** Calcified plaque.

The calculation method for remnant cholesterol (RC) is defined as total cholesterol (TC) minus high-density lipoprotein cholesterol (HDL-C) and low-density lipoprotein cholesterol (LDL-C). The RCLR was calculated as remnant cholesterol (in mmol/L) divided by the lymphocyte percentage expressed as a proportion, that is, RCLR = RC/(lymphocyte percentage/100); for example, a remnant cholesterol of 0.63 mmol/L with a lymphocyte percentage of 30.6% yields an RCLR of 0.63/0.306 = 2.06. Patients were categorized into three groups based on their RCLR values: Group 1 comprised 66 cases with RCLR ≤ 3.03; Group 2 included 68 cases with 3.03 < RCLR ≤ 4.66; and Group 3 consisted of 67 cases with RCLR > 4.66. This study employed the OCT offline workstation (OPTISTM MOBILE SYSTEM) for frequency domain optical coherence tomography, which was performed by two independent observers in the coronary vascular imaging laboratory. The processing of OCT images was conducted by Abbott Medical. During the image analysis, the observers were blinded to the patients' coronary angiography data and clinical symptoms. The analyses and measurements adhered to established OCT standards (18, 19), and a consensus was achieved through comprehensive discussion between the two independent observers, the typical OCT images are shown in Figure 2. In this study, the culprit lesion of the coronary vessel was defined as the region centered on the culprit lesion plaque, extending more than 5 mm on both sides of the normal vascular segment.

### Statistical analysis

Data analysis was conducted using SPSS version 27.0 and Python 3.11 (statsmodels 0.14), while bar charts and box plots were generated with GraphPad Prism version 10.1.2. The normality of quantitative data was assessed using the Kolmogorov–Smirnov test. For quantitative variables with a normal distribution, comparisons among the three groups were performed using one-way analysis of variance, with results presented as mean ± standard deviation (Mean ± SD); for variables that did not follow a normal distribution, the Kruskal–Wallis *H*-test was used, with results expressed as median (interquartile range) [M (P25, P75)]. Qualitative variables were compared using the chi-square test and reported as percentages (%). When an omnibus test was statistically significant, pairwise *post-hoc* comparisons among the three groups were performed with Bonferroni correction; because three pairwise comparisons were possible (Group 1 vs. Group 2, Group 1 vs. Group 3, and Group 2 vs. Group 3), the adjusted significance threshold was set at 0.05/3 ≈ 0.017. To assess whether RCLR was independently associated with the OCT-defined vulnerable plaque features, multivariable logistic regression analyses were performed separately for lipid-rich plaque, TCFA, and plaque rupture. RCLR was analyzed primarily as tertiles (the lowest tertile as reference) and secondarily as a standardized continuous variable (per 1-SD increase); a linear trend was tested by entering the tertile rank as an ordinal variable (1, 2, 3). The primary model was adjusted for age, sex, smoking status, diabetes mellitus, and hypertension, selected *a priori* as clinically relevant confounders while limiting model complexity given the number of events, particularly for TCFA. Variables that mathematically define RCLR (TC, HDL-C, LDL-C, remnant cholesterol, and lymphocyte percentage) were not entered, to avoid overadjustment for the components of the exposure and multicollinearity; in-hospital statin therapy was not entered because nearly all patients received statins after admission, providing insufficient variation for stable estimation (pre-admission statin use was unavailable; see Limitations). Robustness was examined with Firth penalized logistic regression, with additional adjustment for selected non-component metabolic or inflammatory variables (fasting glucose and triglycerides, white blood cell count, neutrophil count, or aspartate aminotransferase), and with modified Poisson regression with robust variance to estimate prevalence ratios. ROC curves were plotted using the qualitative OCT plaque results as the dependent variable to evaluate the predictive value of RCLR. Spearman correlation was used to assess the relationship between RCLR and the quantitative OCT parameters within the lipid-plaque subgroup; this subgroup analysis was exploratory and not pre-specified. A two-sided *P* value < 0.05 was considered statistically significant, except for the Bonferroni-corrected pairwise comparisons (*P* < 0.017).

## Results

### Baseline clinical characteristics grouped according to RCLR

A comparative analysis of the baseline data across the three patient groups was performed, with the results summarized in Table 1. The average age of the 201 patients was 58.8 years (±10.4), with a predominance of males, who comprised 159 cases (79.1%). Among the patients, there were 101 cases of hypertension (50.2%), 49 cases of diabetes (24.4%), and 126 cases with a history of smoking (62.7%). No statistically significant differences were observed among the three groups (*p* > 0.05). In terms of laboratory indicators, statistically significant differences (*P* < 0.05) were noted among the three groups for white blood cell count (WBC), neutrophil count (NEUT#), lymphocyte percentage (LYMPH%), fasting venous blood glucose (FBG), aspartate aminotransferase (AST), TC, TG, and LDL-C. Regarding in-hospital medication, only the use of clopidogrel and ticagrelor showed statistically significant differences (*p* < 0.05), while no significant differences were found in the use of other medications among the three groups (*p* > 0.05).

**Table 1:** Characteristics.

Variable	RCLR Total	RCLR Group			*P*
		1	2	3	
	(*n* = 201)	(*n* = 66)	(*n* = 68)	(*n* = 67)	
Age, years	58.8 (10.4)	59.3 (9.85)	59.5 (10.3)	57.4 (11.0)	0.451
Male, *n* (%)	159 (79.1%)	50 (75.8%)	56 (82.4%)	53 (79.1%)	0.644
Smoker	126 (62.7%)	44 (66.7%)	41 (60.3%)	41 (61.2%)	0.713
Hypertension	101 (50.2%)	34 (51.5%)	34 (50.0%)	33 (49.3%)	0.965
Diabetes mellitus	49 (24.4%)	17 (25.8%)	20 (29.4%)	12 (17.9%)	0.283
Laboratory parameters					
WBC, (10^9^/L)	7.76 (2.58)	6.49 (1.87)	7.42 (2.04)	9.35 (2.86)	<0.001
LYMPH%	26.2 (9.60)	33.7 (6.97)	27.4 (5.93)	17.6 (7.90)	<0.001
NEUT#	5.12 (2.37)	3.71 (1.25)	4.69 (1.62)	6.95 (2.68)	<0.001
FBG, mmol/L	5.43 (4.84–6.47)	5.27 (4.69–5.75)	5.42 (4.83–6.57)	5.64 (5.00–6.66)	0.040
HbA1c, %	6.82 (4.56)	6.35 (1.04)	7.68 (7.60)	6.40 (1.26)	0.260
ALT, U/L	25.0 (17.0–36.0)	25.0 (18.2–33.8)	24.0 (16.0–37.0)	28.0 (17.5–42.5)	0.469
AST, U/L	24.0 (17.0–37.0)	21.0 (17.0–27.5)	22.0 (17.5–32.5)	32.0 (18.5–112)	0.001
Cr, umol/L	68.0 (61.0–79.0)	72.0 (62.0–81.0)	68.0 (60.0–79.0)	68.0 (61.5–77.5)	0.663
TC, mmol/L	4.44 (1.24)	3.62 (0.90)	4.64 (0.87)	5.05 (1.40)	<0.001
TG, mmol/L	1.48 (1.11–2.21)	1.21 (0.84–1.56)	1.78 (1.24–2.31)	1.84 (1.25–2.82)	<0.001
HDL-C, mmol/L	1.02 (0.22)	1.03 (0.24)	1.02 (0.20)	1.00 (0.24)	0.862
LDL-C, mmol/L	2.43 (0.80)	1.89 (0.66)	2.60 (0.63)	2.79 (0.80)	<0.001
LPa, mg/L	240 (208)	230 (196)	221 (218)	269 (211)	0.375
Used during hospitalization					
Aspirin, *n* (%)	186 (92.5%)	61 (92.4%)	64 (94.1%)	61 (91.0%)	0.800
Clopidogrel, *n* (%)	57 (28.4%)	25 (37.9%)	20 (29.4%)	12 (17.9%)	0.037
Ticagrelor, *n* (%)	133 (66.2%)	35 (53.0%)	45 (66.2%)	53 (79.1%)	0.006
Statins, *n* (%)	199 (99.0%)	66 (100%)	68 (100%)	65 (97.0%)	0.217
ACEI/ARB/ARNI, *n* (%)	107 (53.2%)	31 (47.0%)	40 (58.8%)	36 (53.7%)	0.387
*Β*-blockers, *n* (%)	140 (69.7%)	43 (65.2%)	48 (70.6%)	49 (73.1%)	0.593

WBC, white blood cell; FBG, fasting blood glucose; ALT, alanine aminotransferase; AST, aspartate aminotransferase; Cr, creatinine; TC, total cholesterol; TG, triglyceride; HDL-C, high-density lipoprotein cholesterol; LDL-C, low-density lipoprotein cholesterol.

### Comparative analysis of RCLR and coronary angiography

The analysis results comparing RCLR with coronary angiography are presented in Table 2. Among the 201 identified culprit lesions, the left anterior descending artery was the culprit vessel in 114 cases, representing 56.7% of the total. The right coronary artery was identified as the culprit vessel in 59 cases, accounting for 29.4%. Notably, the distribution of culprit vessels in the left anterior descending artery and the right coronary artery exhibited significant differences among the three groups (*p* < 0.05). Regarding the location of the culprit vessels, 109 lesions were found in the proximal segment, constituting 54.2%, with significant differences also observed among the three groups (*p* < 0.05).

**Table 2:** Analysis of RCLR and coronary angiography results.

Variable	RCLR Total	RCLR Group			*P*
		1	2	3	
	(*n* = 201)	(*n* = 66)	(*n* = 68)	(*n* = 67)	
Criminal's blood vessel, %					
LAD	114 (56.7%)	28 (42.4%)	37 (54.4%)	49 (73.1%)	0.002
LCX	25 (12.4%)	11 (16.7%)	9 (13.2%)	5 (7.46%)	0.266
RCA	59 (29.4%)	26 (39.4%)	20 (29.4%)	13 (19.4%)	0.041
D1	3 (1.49%)	1 (1.52%)	2 (2.94%)	0 (0.00%)	0.660
Number of vascular lesions					
Single vessel disease	126 (62.7%)	46 (69.7%)	42 (61.8%)	38 (56.7%)	0.296
Multiple lesions	75 (37.3%)	20 (30.3%)	26 (38.2%)	29 (43.3%)	0.296
Distribution of criminal lesions					
Proximal	109 (54.2%)	30 (45.5%)	33 (48.5%)	46 (68.7%)	0.014
Middle	56 (27.9%)	19 (28.8%)	24 (35.3%)	13 (19.4%)	0.118
Distal	36 (17.9%)	17 (25.8%)	11 (16.2%)	8 (11.9%)	0.104

LAD, left anterior descending artery; LCX, left circumflex artery; RCA, right coronary artery; D1, first diagonal branch.

### Differences in OCT image features Among different RCLR groups

**Figure 3:**
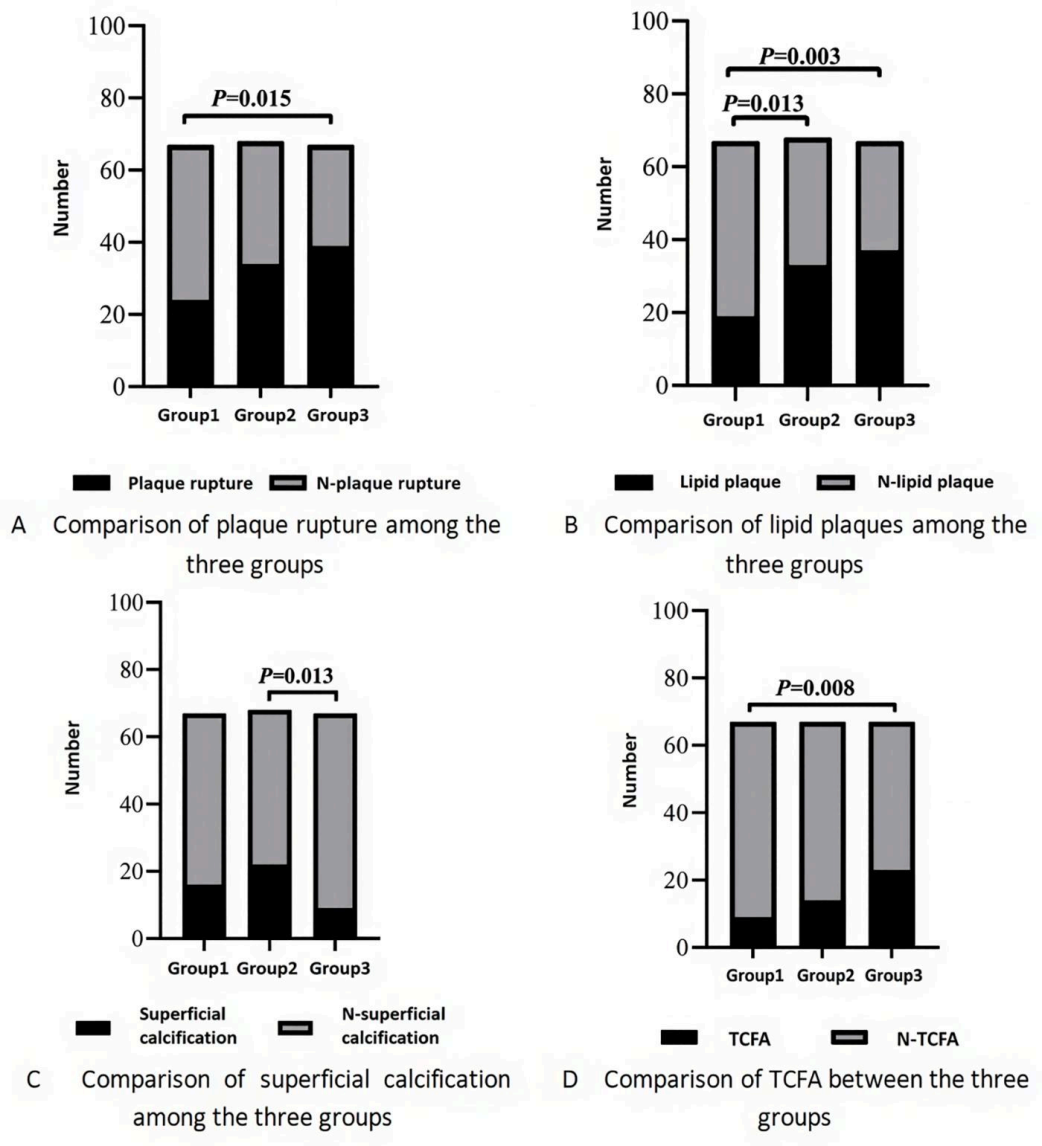
Comparison of plaque-related pathological features among three groups. This figure presents stacked bar charts illustrating the distribution of four plaque pathological characteristics across Group 1, Group 2, and Group 3, with statistical comparisons (*P*-values) labeled for significant differences.

In the qualitative analysis of OCT, statistically significant differences were identified in the composition ratios of plaque rupture, lipid plaque, TCFA, and superficial calcification among the RCLR groups (*p* < 0.05). The intergroup comparison results indicated significant differences in plaque rupture between Group 1 and Group 3 (*p* < 0.017). For lipid plaque, significant differences were noted between Group 1 and Group 2, Group 1 and Group 3, as well as between Group 2 and Group 3 (*p* < 0.017). Regarding superficial calcification, a significant difference was found between Group 2 and Group 3 (*p* < 0.017). Furthermore, a significant difference in TCFA was also observed between Group 1 and Group 3 (*p* < 0.017). Detailed results are presented in Figure 3 and Table 3. These findings highlight significant differences in the qualitative characteristics of lipid plaques observed via OCT among the RCLR groups. In the quantitative analysis of OCT, no statistically significant differences (*p* > 0.05) were observed in lesion length, proximal reference area, distal reference area, minimum lumen area, diameter stenosis, and area stenosis among the RCLR groups.

**Table 3:** The relationship between RCLR and OCT measurements of plaque characteristics.

Variable	RCLR Total	RCLR Group			*P*	*P*		
		1	2	3		1 VS 2	1 VS 3	2 VS 3
	(*n* = 201)	(*n* = 66)	(*n* = 68)	(*n* = 67)				
Qualitative analysis								
Rupture	97 (48.3%)	24 (36.4%)	34 (50.0%)	39 (58.2%)	0.039	0.118	0.015	0.389
Erosion	21 (10.4%)	4 (6.06%)	7 (10.3%)	10 (14.9%)	0.247			
Fibrous	172 (85.6%)	54 (81.8%)	63 (92.6%)	55 (82.1%)	0.124			
Lipid rich	89 (44.3%)	19 (28.8%)	33 (48.5%)	37 (55.2%)	0.006	0.013	0.003	0.465
Chelesterol crystal	35 (17.4%)	8 (12.1%)	12 (17.6%)	15 (22.4%)	0.295			
Microchannel	54 (26.9%)	24 (36.4%)	12 (17.6%)	18 (26.9%)	0.051			
Macrophage	185 (92.0%)	60 (90.9%)	61 (89.7%)	64 (95.5%)	0.421			
Calcification	88 (43.8%)	30 (45.5%)	33 (48.5%)	25 (37.3%)	0.399			
Superficial microcalcification	47 (23.4%)	16 (24.2%)	22 (32.4%)	9 (13.4%)	0.034	0.183	0.091	0.013
Spotty calcification	45 (22.4%)	16 (24.2%)	14 (20.6%)	15 (22.4%)	0.879			
TCFA	46 (23.0%)	9 (13.6%)	14 (20.9%)	23 (34.3%)	0.016	0.360	0.008	0.122
Quantitative analysis								
Length of lesion, mm	15.0 (11.4–19.1)	13.8 (10.0–17.5)	14.6 (11.4–19.7)	16.1 (12.4–19.8)	0.262			
Measuring the lumen area of the proximal reference tube, mm²	9.38 (7.09–11.9)	9.57 (7.03–11.6)	9.32 (6.75–11.8)	9.33 (7.22–12.7)	0.597			
Measuring the lumen area of the distal reference tube, mm²	7.06 (5.68–9.50)	7.30 (5.80–9.11)	7.17 (5.24–9.22)	6.67 (5.77–9.91)	0.985			
Minimum lumen area, mm²	1.92 (1.34–2.84)	2.04 (1.54–2.80)	1.82 (1.19–2.50)	1.76 (1.32–3.04)	0.210			
The degree of stenosis in diameter, %	50.8 (43.8–58.9)	50.0 (40.6–56.7)	51.5 (44.2–60.0)	50.1 (45.7–58.5)	0.409			
Extent of area narrowing, %	75.7 (68.3–83.0)	73.4 (65.7–81.4)	76.6 (68.7–84.1)	75.3 (70.0–82.3)	0.366			

Pairwise comparisons among the three groups were performed using the chi-square or Kruskal–Wallis test with Bonferroni correction; the adjusted significance threshold was *P* < 0.05/3 ≈ 0.017.

### The association between RCLR and plaque characteristics in OCT images

**Figure 4:**
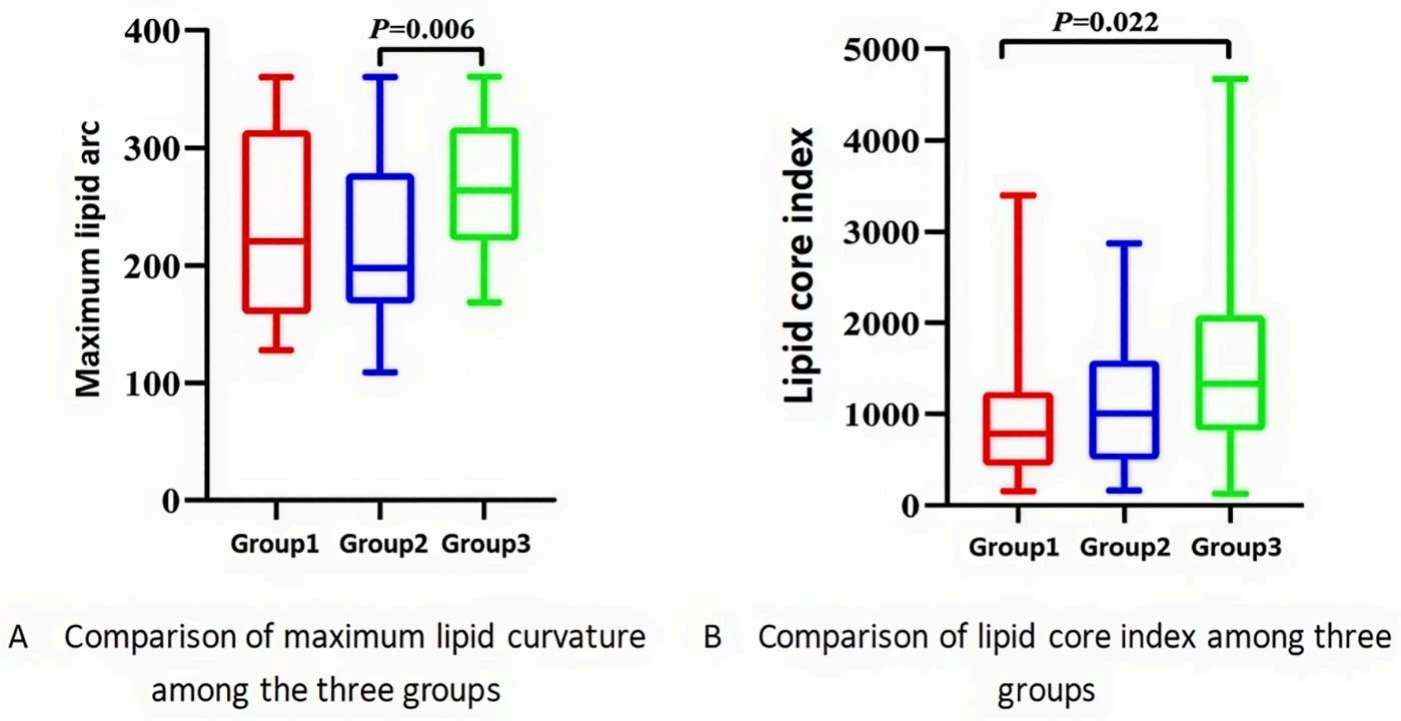
Comparison of lipid-related plaque parameters among three groups. This figure displays box-and-whisker plots (with red/blue/green representing Group 1/2/3, respectively) that compare two lipid-associated plaque metrics across three groups, with significant *P*-values labeled.

This study subsequently explored the relationship between RCLR and the quantitative parameters of OCT lipid plaques in 89 cases, as presented in Table 4. Among these parameters, the maximum lipid arc and lipid index exhibited statistically significant differences among the three groups (*p* < 0.05). For the indicators that demonstrated statistical significance, pairwise comparisons were performed with Bonferroni correction (adjusted *P* < 0.05, equivalent to an unadjusted threshold of *P* < 0.017). The results revealed a statistically significant difference in the maximum lipid arc between Group 2 and Group 3 (*p* < 0.05); similarly, a statistically significant difference was observed in the lipid index between Group 1 and Group 3 (*p* < 0.05). This suggests a correlation between RCLR and the quantitative parameters of lipid plaques, as detailed in Table 4, Figure 4. This subgroup analysis was restricted to the 89 culprit lesions containing lipid plaque and was exploratory rather than pre-specified.

**Table 4:** The relationship between RCLR and quantitative data of lipid plaque measured by OCT.

Variable	RCLR Total	RCLR Group			*P*	*P*		
		1	2	3		1 VS 2	1 VS 3	2 VS 3
	(*n* = 89)	(*n* = 19)	(*n* = 33)	(*n* = 37)				
Minial FCT, um	60.0 (50.0–80.0)	60.0 (40.0–92.5)	65.0 (50.0–100)	50.0 (50.0–65.0)	0.080			
Avreage FCT, um	92.2 (70.0–136)	95.0 (75.8–124)	99.6 (78.0–148)	83.3 (65.3–117)	0.224			
Maximal lipid arc,°	244 (183–301)	221 (164–314)	198 (171–273)	264 (229–319)	0.005	1.000	0.196	0.006
Mean lipid-rich arc,°	170 (138–214)	157 (120–191)	167 (137–209)	181 (149–236)	0.207			
Lipid core length, mm	6.50 (3.80–9.55)	6.10 (2.95–8.20)	6.00 (3.80–9.03)	8.30 (5.35–10.8)	0.144			
Lipid core index	1,102 (619–1,744)	787 (434–1,227)	1,007 (560–1,557)	1,333 (883–2,057)	0.047	0.478	0.022	0.066

Pairwise comparisons were performed with Bonferroni correction (adjusted *P* < 0.05, equivalent to an unadjusted threshold of *P* < 0.017).

**Figure 5:**
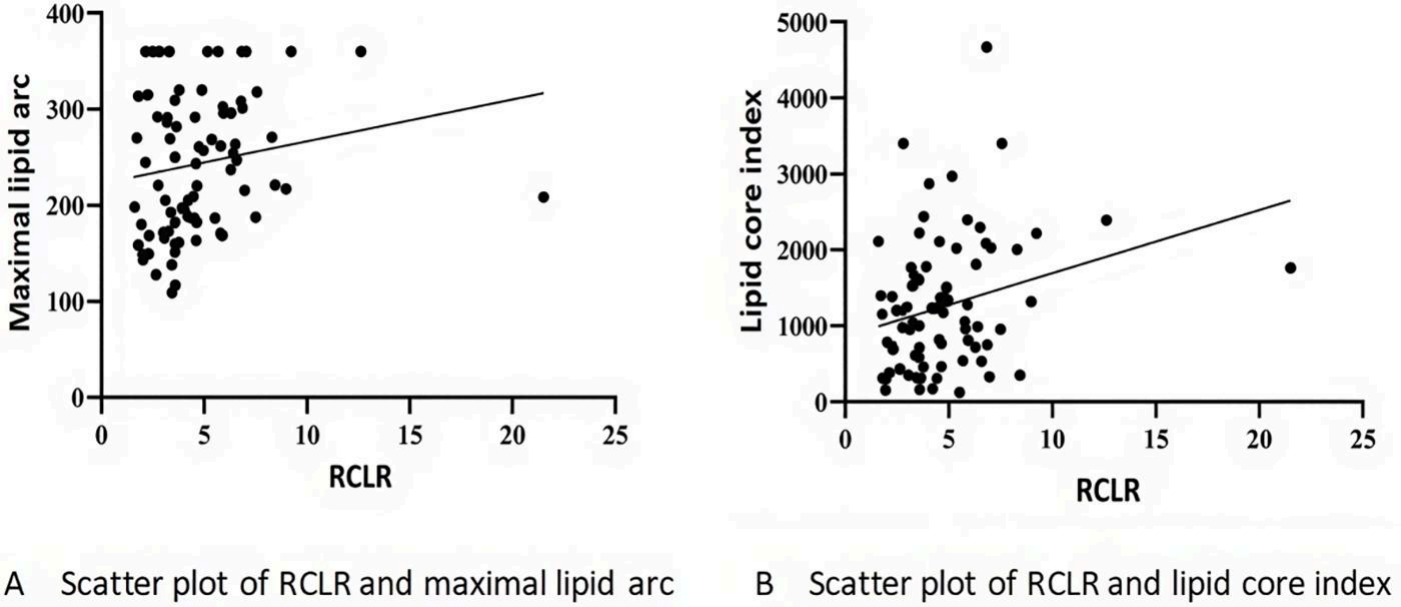
Correlation analysis between RCLR and lipid-related plaque metrics. This figure presents scatter plots illustrating the relationships between the RCLR (abbreviation to be defined) and two lipid-associated plaque parameters.

Spearman correlation analysis was performed to further examine the relationship between RCLR and the quantitative OCT parameters within the lipid-plaque subgroup (Table 5). RCLR showed weak but statistically significant positive correlations with the maximum lipid arc (*r* = 0.252) and the lipid index (*r* = 0.279), with higher RCLR values corresponding to a larger maximum lipid arc and lipid index (Figure 5). Given the modest magnitude of these coefficients, these correlations should be interpreted with caution and regarded as hypothesis-generating rather than indicative of a strong quantitative relationship.

**Table 5:** Linear correlation analysis between RCLR and quantitative plaque characteristics under OCT.

Quantitative parameters of lipid plaque	correlation coefficient	*P*	95%CI
Maximal lipid arc,°	0.252	0.022	0.026–0.454
Lipid core index	0.279	0.011	0.062–0.481

**Figure 6:**
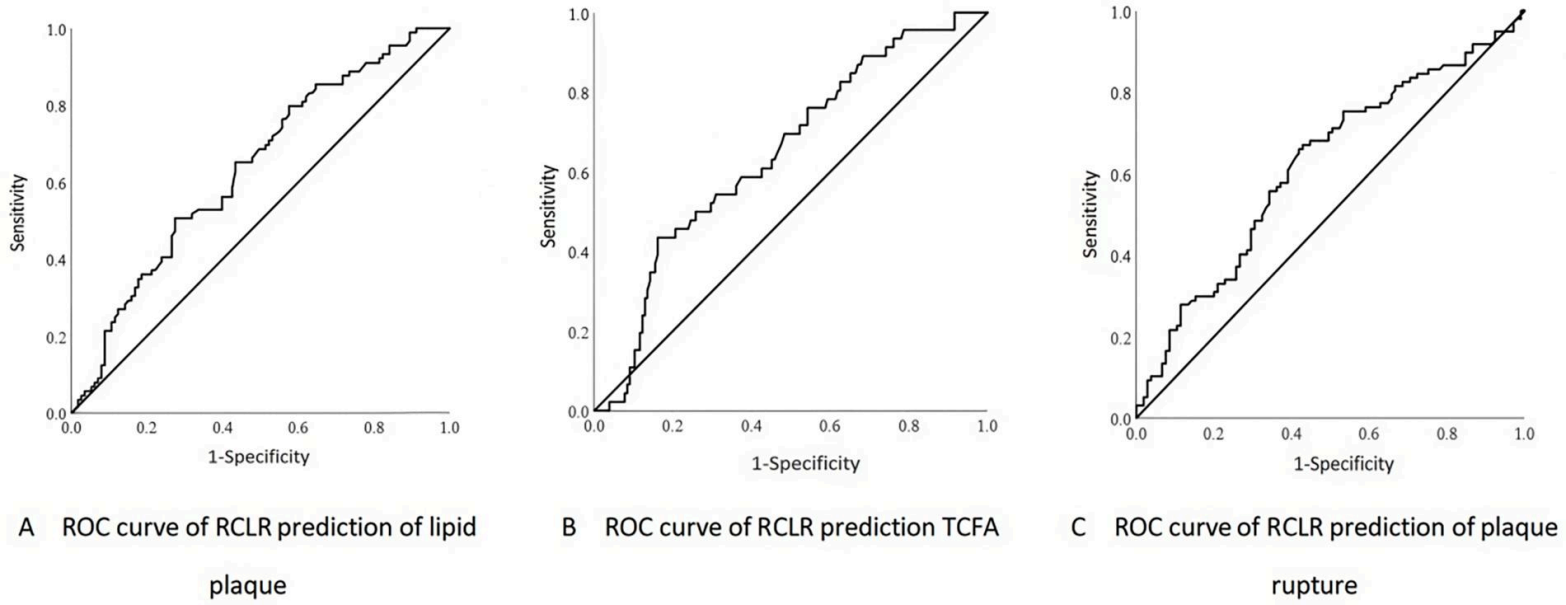
ROC curves for RCLR in predicting plaque-related phenotypes.

The ROC curves for predicting the qualitative characteristics of OCT plaques using RCLR were plotted. The results indicated that RCLR possesses significant predictive capabilities for lipid plaques, TCFA, and plaque rupture (*p* < 0.05), as illustrated in Figure 6. The optimal cutoff values for RCLR in predicting lipid plaques, TCFA, and plaque rupture were determined to be 4.415, 5.795, and 3.490, respectively, as presented in Table 6.

**Table 6:** Predictive value of RCLR for qualitative features of OCT plaques.

Plaque characteristics	AUC	95%CI	Best cutoff value	Sensitivity, %	Specificity,%	Youden index	*P*
Lipid rich	0.635	0.559–0.712	4.415	50.6	72.6	0.231	0.001
TCFA	0.646	0.559–0.733	5.795	43.5	83.9	0.273	0.003
Rupture	0.618	0.540–0.696	3.490	67.0	57.1	0.242	0.004

**Figure 7:**
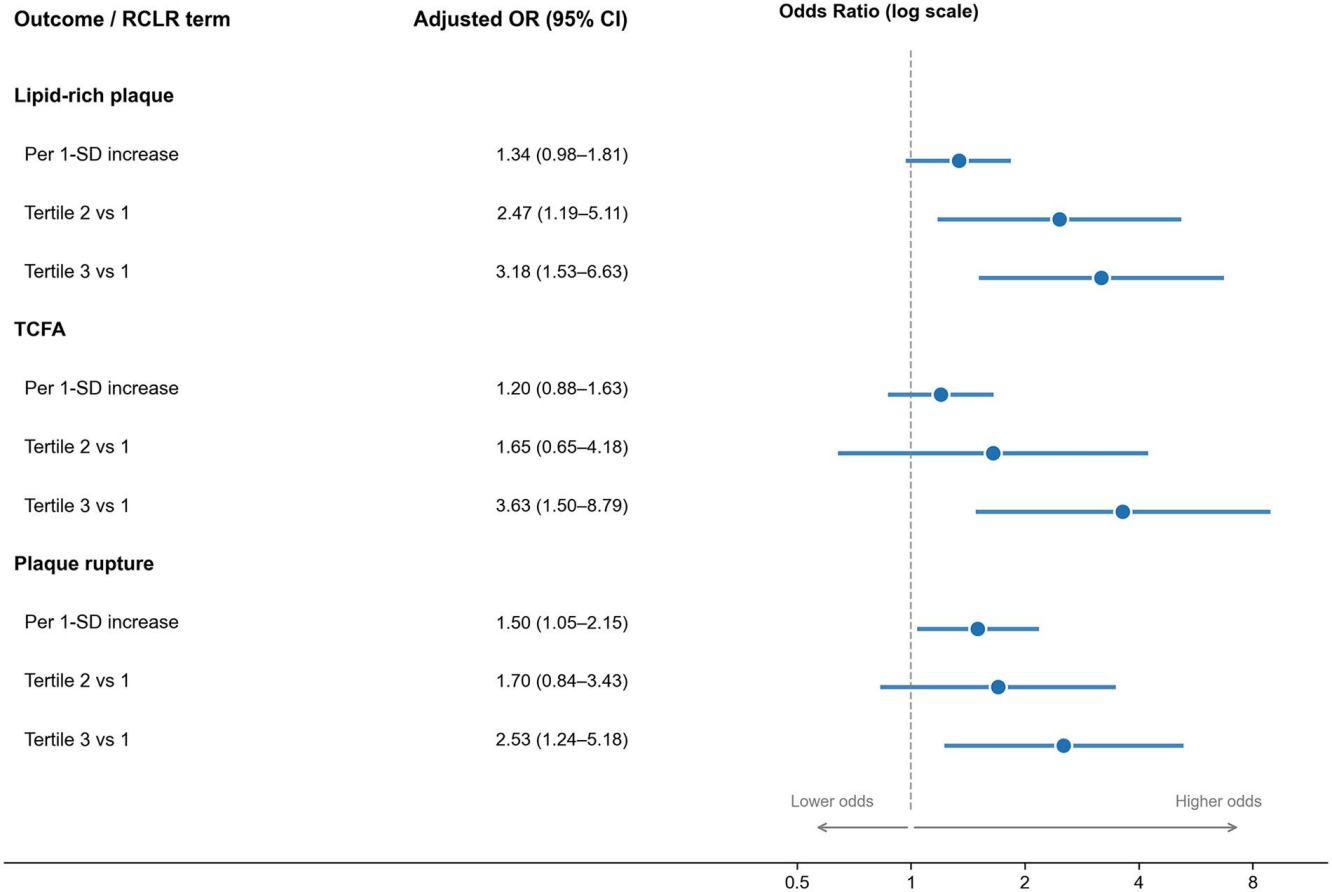
Forest plot of the multivariable-adjusted associations between RCLR and OCT-defined vulnerable plaque features. Squares denote adjusted odds ratios and horizontal lines the 95% confidence intervals, for each 1-SD increase in RCLR and for RCLR tertiles 2 and 3 vs. tertile 1; models were adjusted for age, sex, smoking status, diabetes mellitus, and hypertension. RCLR, remnant cholesterol to lymphocyte ratio; OCT, optical coherence tomography; TCFA, thin-cap fibroatheroma.

In multivariable logistic regression, a higher RCLR was associated with the OCT-defined vulnerable plaque features after adjustment for age, sex, smoking status, diabetes mellitus, and hypertension (Table 7, Figure 7). Compared with the lowest tertile, the highest RCLR tertile was associated with greater odds of lipid-rich plaque (adjusted OR 3.18, 95% CI 1.53–6.63), TCFA (adjusted OR 3.63, 95% CI 1.50–8.79), and plaque rupture (adjusted OR 2.53, 95% CI 1.24–5.18), with a significant graded trend across tertiles (all P for trend ≤0.011). When RCLR was modelled as a continuous variable, each 1-SD increase was associated with plaque rupture (adjusted OR 1.50, 95% CI 1.05–2.15), whereas the per-SD associations with lipid-rich plaque and TCFA did not reach significance, indicating a graded rather than strictly linear relationship. These associations were directionally consistent in Firth penalized logistic regression and in modified Poisson models estimating prevalence ratios, and remained robust after additional adjustment for fasting glucose and triglycerides or for aspartate aminotransferase (Supplementary Table S2). The associations were attenuated, however, after further adjustment for white blood cell or neutrophil count—particularly for plaque rupture—indicating that part of the signal captured by RCLR overlaps with leukocyte inflammatory parameters that are intrinsically related to its lymphocyte-percentage denominator (Supplementary Table S2).

**Table 7:** Crude and multivariable-adjusted associations between RCLR and OCT-defined vulnerable plaque features.

Outcome	RCLR	Crude OR (95% CI)	P	Adjusted OR (95% CI)^a^	P
Lipid-rich plaque (events=89/201)	Per 1-SD increase	1.33 (0.98–1.81)	0.072	1.34 (0.98–1.81)	0.063
	Tertile 1 (reference)	1.00	—	1.00	—
	Tertile 2	2.33 (1.14–4.76)	0.020	2.47 (1.19–5.11)	0.015
	Tertile 3	3.05 (1.49–6.26)	0.002	3.18 (1.53–6.63)	0.002
	P for trend				0.002
TCFA (events=46/201)	Per 1-SD increase	1.16 (0.86–1.56)	0.341	1.20 (0.88–1.63)	0.243
	Tertile 1 (reference)	1.00	—	1.00	—
	Tertile 2	1.64 (0.66–4.11)	0.289	1.65 (0.65–4.18)	0.288
	Tertile 3	3.31 (1.39–7.86)	0.007	3.63 (1.50–8.79)	0.004
	P for trend				0.003
Plaque rupture (events=97/201)	Per 1-SD increase	1.47 (1.05–2.08)	0.027	1.50 (1.05–2.15)	0.025
	Tertile 1 (reference)	1.00	—	1.00	—
	Tertile 2	1.75 (0.88–3.49)	0.112	1.70 (0.84–3.43)	0.139
	Tertile 3	2.44 (1.21–4.90)	0.012	2.53 (1.24–5.18)	0.011
	P for trend				0.011

RCLR, remnant cholesterol to lymphocyte ratio; OCT, optical coherence tomography; OR, odds ratio; CI, confidence interval; TCFA, thin-cap fibroatheroma.

a Adjusted for age, sex, smoking status, diabetes mellitus, and hypertension.

RCLR tertiles: T1 ≤ 3.03; T2 3.03–4.66; T3 > 4.66. Per 1-SD increase corresponds to one standard deviation of RCLR. P for trend was obtained by modelling tertile rank as an ordinal variable (1, 2, 3) in the adjusted model.

## Discussion

To our knowledge, no studies have yet investigated the correlation between the RCLR and the characteristics of coronary plaques in patients with ACS. The main findings of this study are as follows: a) Higher RCLR values are significantly associated with increased incidences of TCFA, plaque rupture, and lipid index in culprit lesions, indicating enhanced plaque instability; b) RCLR demonstrates a significant correlation with lipid-rich plaques, TCFA, and plaque rupture in ACS patients, and exhibits predictive capacity for these indicators. c) These associations persisted in multivariable analysis, in which the highest RCLR tertile remained associated with lipid-rich plaque, TCFA, and plaque rupture after adjustment for major clinical risk factors, although they were partly attenuated after additional adjustment for leukocyte inflammatory parameters.

The association between elevated levels of RCLR and a higher incidence of MACE is biologically plausible. RC is a component of triglyceride-rich lipoproteins (TRLs), which can traverse the arterial wall and be absorbed by macrophages and smooth muscle cells. During the intracellular metabolism of TRLs, triglycerides are degraded, releasing cholesterol. The undegraded cholesterol then accumulates around the arterial wall, ultimately forming atherosclerotic plaques (20–22). This mechanism of plaque formation may elucidate the significant association between RC and MACE. A low lymphocyte count and a high neutrophil count are typical inflammatory responses linked to the progression of atherosclerotic plaques and stent implantation (23–25). Additionally, RC can induce the production of cytokines and interleukins through plasminogen activator inhibitor-1 (26), thereby triggering an inflammatory response. Previous studies have demonstrated an association between higher levels of RC and severe cardiovascular diseases. Jepsen et al. (27) reported that, after following a cohort of ischemic heart disease patients for over 35,000 person-years, high levels of RC (but not low-density lipoprotein cholesterol, LDL-C) were associated with increased all-cause mortality. Similarly, a retrospective study by Varbo et al. (28) showed that for every 1 mmol/L increase in RC, the risk of ischemic heart disease increased by 2.8 times, independent of the additional risk associated with decreased levels of HDL-C. A multicenter randomized clinical trial involving an elderly cohort further confirmed that higher RC levels were associated with an increased risk of MACE (29). In addition to a high RC, studies have demonstrated an association between reduced lymphocyte counts and an increased incidence of adverse events in patients with cardiovascular diseases (30). In summary, the RCLR, by integrating the dual effects of blood lipids and inflammation, serves as a reliable indicator for predicting MACE. Furthermore, this analysis of the correlation between RCLR and OCT plaque has further clarified the relationship between RCLR and MACE.

The pathogenesis of ACS is primarily associated with the abrupt formation of thrombus within the coronary arteries, leading to significant narrowing or complete occlusion of the vascular lumen (31). Existing pathological studies indicate that plaque rupture accounts for 60%, plaque erosion for 35%, and calcified nodules for 5% of the primary causes of acute lesions in ACS (31). The primary pathological characteristics of coronary vulnerable plaques include a large lipid-rich necrotic core, a thin fibrous cap, and macrophage infiltration, a conclusion thoroughly substantiated by autopsy-related studies (32). Furthermore, relevant research has demonstrated that TCFA, macrophage infiltration, microchannels, cholesterol crystallization, and spotty calcification are significant manifestations of vulnerable plaques, as observed through OCT (33, 34). Our study reveals that ACS patients in the high RCLR group exhibit significantly higher rates of lipid plaques, TCFA, and elevated lipid indices compared to the other groups. This finding may elucidate, from the perspective of plaque stability, the underlying reasons for the increased incidence of MACE in patients with a high RCLR.

The association between RCLR and vulnerable plaque features that persisted in multivariable analysis argues against the possibility that the univariable group differences were driven solely by baseline imbalances in metabolic and inflammatory parameters. Notably, the attenuation observed after additional adjustment for white blood cell or neutrophil count is biologically plausible and informative. RCLR was constructed as a composite index integrating remnant-cholesterol burden and lymphocyte-percentage–based inflammatory status; leukocyte-differential parameters are therefore not purely external confounders but partly represent overlapping inflammatory information embedded in the denominator of RCLR. Accordingly, the present findings should not be interpreted as evidence that RCLR is independent of the leukocyte inflammatory milieu. Rather, RCLR appears to reflect a combined lipid–inflammatory phenotype associated with OCT-defined plaque vulnerability, an interpretation supported by the persistence of associations in the primary clinical model, in Firth penalized and modified Poisson models, and in the metabolic sensitivity analyses. Among the three features, the plaque-rupture association was the most sensitive to additional inflammatory-cell adjustment, whereas the lipid-rich plaque association was the most robust.

This study has several limitations. First, as a single-center, retrospective, cross-sectional study with a modest sample size, residual confounding cannot be excluded and a temporal or causal relationship between RCLR and plaque characteristics cannot be established. Second, 149 patients were excluded because of poor OCT image quality, which could introduce selection bias; however, the included and the excluded patients did not differ significantly in any baseline demographic, laboratory, or medication variable (Supplementary Table S1), which reduces concern for major measured baseline imbalance; nevertheless, selection bias from unmeasured factors cannot be excluded. Third, prior medication history before admission—particularly pre-admission statin therapy, which can influence both lipid parameters and plaque composition on OCT—was not available and could not be accounted for. Fourth, although the highest RCLR tertile remained associated with vulnerable plaque features after adjustment for major clinical risk factors, the associations were partly attenuated after additional adjustment for white blood cell or neutrophil count, indicating that the information carried by RCLR overlaps with systemic inflammatory-cell parameters; RCLR should therefore be regarded as a composite lipid–inflammatory marker rather than one fully independent of leukocyte inflammation. Fifth, the quantitative lipid-plaque analyses were restricted to the subgroup of culprit lesions containing lipid plaque and were exploratory and underpowered. Future multi-center, large-sample, prospective studies are needed to clarify the relationship between RCLR and lipid plaque, plaque rupture, and TCFA under OCT.

## Conclusion

RCLR is correlated with lipid plaques, TCFA, and plaque rupture in ACS patients as observed through OCT, and the highest RCLR tertile was associated with these features after adjustment for major clinical risk factors. A higher RCLR indicates an increased potential vulnerability of the plaque, suggesting that RCLR may serve as a composite lipid–inflammatory marker of coronary plaque vulnerability; because its association partly overlaps with leukocyte inflammatory parameters, however, RCLR should not be regarded as independent of systemic inflammation.

## Data Availability

The original contributions presented in the study are included in the article/Supplementary Material, further inquiries can be directed to the corresponding author.
