# Supplementary material for: Higher remnant cholesterol to lymphocyte ratio is associated with vulnerable plaque features in acute coronary syndrome
Source: Front Cardiovasc Med. 2026 Jul 23;13:1785669. doi: 10.3389/fcvm.2026.1785669 (PMC13441940; doi:10.3389/fcvm.2026.1785669)
Supplement: Supplementary file 1 [file Table1.docx]

Table S1 Characteristics of the excluded and included participants

|  | Included  （n=201） | Excluded  （n=149） | *P* |
| --- | --- | --- | --- |
| Age, years | 58.8 （10.4） | 59.0 (10.6) | 0.736 |
| Male，n（%） | 159 （79.1%） | 115 （77.2%） | 0.746 |
| Hypertension | 101 （50.2%） | 75 （50.3%） | >0.999 |
| Diabetes mellitus | 49 （24.4%） | 41 （27.5%） | 0.570 |
| Laboratory parameters |  |  |  |
| WBC，（10^9^/L） | 7.76 （2.58） | 7.67 （2.53） | 0.682 |
| LYMPH% | 26.2 （9.60） | 26.0 (9.70) | 0.798 |
| NEUT# | 5.12 （2.37） | 5.04 (2.35) | 0.752 |
| FBG，mmol/L | 5.43 (4.84-6.47) | 5.45 (4.89-6.56) | 0.664 |
| HbA1c,% | 6.82 （4.56） | 6.95 (5.12) | 0.822 |
| ALT，U/L | 25.0 (17.0-36.0) | 24.0 (16.0-35.0) | 0.544 |
| AST,U/L | 24.0 (17.0-37.0) | 22.0 (17.0-34.0) | 0.652 |
| Cr,umol/L | 68.0 (61.0-79.0) | 68.0 (60.0-79.0) | 0.662 |
| TC，mmol/L | 4.44 (1.24) | 4.42 (1.31) | 0.938 |
| TG，mmol/L | 1.48 (1.11-2.21) | 1.45 (1.10-2.21) | 0.916 |
| HDL-C，mmol/L | 1.02 （0.22） | 1.03 （0.24） | 0.862 |
| LDL-C，mmol/L | 2.43 （0.80） | 2.42 （0.83） | 0.996 |
| LPa，mg/L | 240 （208） | 249 （223） | 0.674 |
| Used during hospitalization |  |  |  |
| Aspirin，n（%） | 186 （92.5%） | 144 （96.6%） | 0.164 |
| Clopidogrel，n（%） | 57 （28.4%） | 46 （30.9%） | 0.674 |
| Ticagrelor，n（%） | 133 （66.2%） | 94 （63.1%） | 0.820 |
| Statins，n（%） | 199 （99.0%） | 142（95.3%） | 0.211 |
| ACEI/ARB/ARNI，n（%） | 107 （53.2%） | 85 （57.0%） | 0.577 |
| Β-blockers，n（%） | 140 （69.7%） | 107 （71.8%） | 0.493 |

Abbreviation：WBC, white blood cell；FBG，fasting blood glucose；ALT, alanine aminotransferase; AST, aspartate aminotransferase;Cr，creatinine；TC，total cholesterol；TG，triglyceride；HDL-C，high-density lipoprotein cholesterol；LDL-C，low-density lipoprotein cholesterol
