# Supplementary material for: Higher remnant cholesterol to lymphocyte ratio is associated with vulnerable plaque features in acute coronary syndrome
Source: Front Cardiovasc Med. 2026 Jul 23;13:1785669. doi: 10.3389/fcvm.2026.1785669 (PMC13441940; doi:10.3389/fcvm.2026.1785669)
Supplement: Supplementary file 2 [file Table2.docx]

Table S2 Sensitivity analyses for the association between RCLR and OCT-defined vulnerable plaque features

| **Outcome** | **Model** | **RCLR per 1-SD‡** | **RCLR Tertile 3 vs 1** | **n** |
| --- | --- | --- | --- | --- |
| Lipid-rich plaque | Primary model (adjusted)§ | 1.34 (0.98–1.81) | 3.18 (1.53–6.63) | 201 |
|  | Firth penalized logistic | 1.31 (0.97–1.76) | 3.04 (1.46–6.31) | 201 |
|  | + Fasting glucose + triglycerides | 1.38 (0.99–1.92) | 3.86 (1.75–8.52) | 195 |
|  | + AST | 1.29 (0.92–1.81) | 3.06 (1.41–6.63) | 200 |
|  | + WBC count | 1.16 (0.85–1.59) | 2.23 (0.99–5.03) | 200 |
|  | + Neutrophil count | 1.08 (0.77–1.52) | 2.01 (0.84–4.80) | 200 |
|  | Modified Poisson (PR) | 1.14 (1.01–1.29) | 1.93 (1.24–2.98) | 201 |
| TCFA | Primary model (adjusted)§ | 1.20 (0.88–1.63) | 3.63 (1.50–8.79) | 201 |
|  | Firth penalized logistic | 1.20 (0.89–1.63) | 3.38 (1.43–8.03) | 201 |
|  | + Fasting glucose + triglycerides | 1.29 (0.94–1.78) | 5.79 (2.17–15.49) | 195 |
|  | + AST | 1.08 (0.76–1.53) | 3.25 (1.28–8.23) | 200 |
|  | + WBC count | 1.02 (0.70–1.48) | 2.74 (1.03–7.25) | 200 |
|  | + Neutrophil count | 0.94 (0.61–1.44) | 2.75 (0.98–7.74) | 200 |
|  | Modified Poisson (PR) | 1.14 (0.96–1.36) | 2.65 (1.31–5.32) | 201 |
| Plaque rupture | Primary model (adjusted)§ | 1.50 (1.05–2.15) | 2.53 (1.24–5.18) | 201 |
|  | Firth penalized logistic | 1.45 (1.03–2.05) | 2.44 (1.20–4.98) | 201 |
|  | + Fasting glucose + triglycerides | 1.81 (1.16–2.81) | 3.63 (1.65–8.02) | 195 |
|  | + AST | 1.35 (0.93–1.95) | 2.06 (0.96–4.40) | 200 |
|  | + WBC count | 1.39 (0.96–2.01) | 2.15 (0.97–4.75) | 200 |
|  | + Neutrophil count | 1.25 (0.86–1.81) | 1.65 (0.70–3.87) | 200 |
|  | Modified Poisson (PR) | 1.14 (1.05–1.24) | 1.61 (1.11–2.34) | 201 |

RCLR, remnant cholesterol to lymphocyte ratio; OCT, optical coherence tomography; OR, odds ratio; PR, prevalence ratio; CI, confidence interval; TCFA, thin-cap fibroatheroma; AST, aspartate aminotransferase; WBC, white blood cell.

All values are OR (95% CI) unless noted; the modified Poisson row reports PR (95% CI) from log-Poisson regression with robust variance.

‡ Per 1 standard deviation increase in RCLR. § Adjusted for age, sex, smoking status, diabetes mellitus, and hypertension.

Each “+ variable” row adds the indicated non-component covariate(s) to the primary model. Variables that mathematically define RCLR (TC, HDL-C, LDL-C, remnant cholesterol, lymphocyte percentage) and statin use (received by 199/201 patients) were not entered. Models are complete-case; n indicates the analysed sample.
